# Differences in the serum metabolome profile of dairy cows according to the BHB concentration revealed by proton nuclear magnetic resonance spectroscopy (^1^H-NMR)

**DOI:** 10.1038/s41598-022-06507-x

**Published:** 2022-02-15

**Authors:** Anastasia Lisuzzo, Luca Laghi, Vanessa Faillace, Chenglin Zhu, Barbara Contiero, Massimo Morgante, Elisa Mazzotta, Matteo Gianesella, Enrico Fiore

**Affiliations:** 1 Department of Animal Medicine, Production and Health (MAPS), University of Padua, Viale dell’Università 16, 35020 Legnaro, Italy; 2 Department of Agricultural and Food Sciences, University of Bologna, 47521 Cesena, Italy; 3 College of Food Science and Technology, Southwest Minzu University, Chengdu, Sichuan China

**Keywords:** Animal physiology, Diagnostic markers

## Abstract

The mobilization of body reserves during the transition from pregnancy to lactation might predispose dairy cows to develop metabolic disorders such as subclinical ketosis or hyperketonemia. These conditions are not easily identifiable and are frequently related to other diseases that cause economic loss. The aim of this study was to evaluate the serum metabolome differences according to the β-hydroxybutyrate (BHB) concentration. Forty-nine Holstein Friesian dairy cows were enrolled between 15 and 30 days in milk. According to their serum BHB concentration, the animals were divided into three groups: Group 0 (G0; 12 healthy animals; BHB ≤ 0.50 mmol/L); Group 1 (G1; 19 healthy animals; 0.51 ≤ BHB < 1.0 mmol/L); and Group 2 (G2; 18 hyperketonemic animals; BHB ≥ 1.0 mmol/L). Animal data and biochemical parameters were examined with one-way ANOVA, and metabolite significant differences were examined by t-tests. Fifty-seven metabolites were identified in the serum samples. Thirteen metabolites showed significant effects and seemed to be related to the mobilization of body reserves, lipids, amino acid and carbohydrate metabolism, and ruminal fermentation.

## Introduction

During the transition period, the higher nutrient demands for fetal growth and milk production cause important metabolic adjustments to support energy requirements^1,2^. In parallel, a reduction in dry matter intake is established (DMI), leading to a negative energy balance (NEB)^3^. The main body reserves mobilized to support energy requirements and glucose synthesis are muscular and adipose tissues^4^. Nonesterified fatty acids (NEFA) are derived from the mobilization of adipose tissue and follow four different energy production pathways in the liver: (*a*) complete oxidation via the tricarboxylic acid cycle (TCA); (*b*) incomplete oxidation to generate ketone bodies and energy; (*c*) export from the liver in the form of very low-density lipoprotein (VLDL) or (*d*) esterification to triacylglycerols and accumulation within hepatocytes^5,6^.

However, the liver cannot metabolize the entire amount of NEFA derived from reserve deployment, generating an excess of ketone bodies (β-hydroxybutyrate (BHB), acetoacetate and acetone). This makes dairy cows more susceptible to metabolic diseases such as ketosis^7^. Ketosis is characterized by an increase in ketone bodies in the blood, urine and milk, which often occurs during early lactation^8^. The β-hydroxybutyrate concentration is used for diagnosis in high-yielding dairy cows, with a cutoff of blood BHB above 1.0–1.4 mmol/L for subclinical ketosis or hyperketonemia without clinical signs^9–11^. The presence of this disease is associated with economic losses due to the risk of abomasum dislocation, reproduction disorders, infectious disease, reduction in milk production and a higher risk of culling animals^9,11^.

Metabolomics is an analytical approach that aims to simultaneously measure the entire metabolite profile of a biologic sample^12^. The metabolites represent the final product of cellular processes in response to environmental changes^13^, so their evaluation could offer a close and complete view of how dairy cows react to metabolic stress in the transition period^14^. Among the analytical tools used for metabolomics investigations, proton nuclear magnetic resonance spectroscopy (^1^H-NMR) requires minimal sample preparation and provides robust and highly reproducible information^12^, which compensates for its lower sensitivity in comparison to other analytical platforms. Serum is a common biological fluid used in this type of analysis because it is easy to sample and minimizes animal welfare problems. Moreover, it can provide metabolites from all organs^15^ to associate them with metabolic disease^16^.

Progressive changes in the metabolomic status of animals during hyperketonemia are measurable. The aim of this study was to evaluate the serum metabolome analyzed using ^1^H-NMR in dairy cows with different levels of BHB.

## Results

### Main characteristics

The serum BHB and NEFA values presented significant differences among each of the groups analyzed (Table 1). In detail, the G1-G0 difference in BHB was 22% of the G2-G0 difference. Focusing on NEFA, Groups G0, G1 and G2 showed mean values of 0.23, 0.34 and 0.62 mEq/L, respectively, so that the G1-G0 difference was 35.9% of the G2-G0 difference.

**Table 1:** Least square means and standard error of the mean (SEM) of main characteristics for each group.

Parameters	G0	G1	G2	SEM	*p* value
BHB (mmol/L)	0.41^c^	0.61^b^	1.43^a^	0.14	< 0.0001
NEFA (mEq/L)	0.23^c^	0.34^b^	0.62^a^	0.08	< 0.01
Glucose (mg/dL)	59.50	63.00	56.50	3.01	NS
DIM (days)	25.80	21.40	21.30	3.44	NS
BCS	2.75	2.75	2.88	0.06	NS
Parity	2.67	2.80	3.00	0.53	NS
Milk yield (kg/day)	28.30	31.30	26.70	2.44	NS

*BHB* β-hydroxybutyrate, *NEFA * nonesterified fatty acid, *DIM* days in milk, *BCS * body condition score, *NS * not significant.

^a-c^Mean values in the same row which differ significantly (*p* value < 0.05).

### Serum metabolome profile and robust principal component analysis (rPCA)

**Figure 1:**
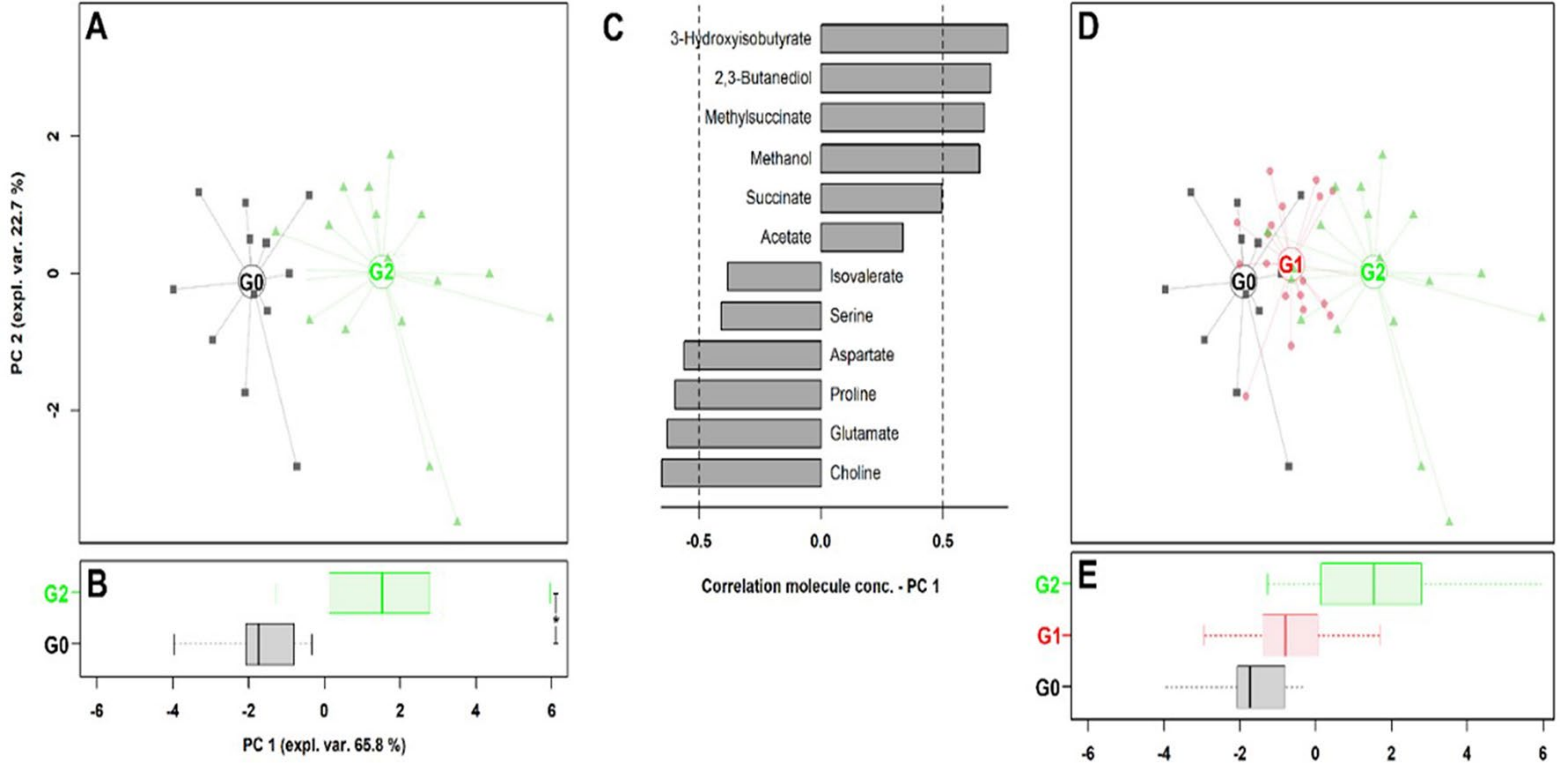
rPCA model built on the space constituted by the concentration of the significant metabolites listed in Table 2 for Groups G0 and G2. In the scoreplot **(A)**, samples from Groups G0 and G2 are represented with black squares and green triangles, respectively. The wide, empty circles represent the median of each sample group. The position of the samples along PC1 is summarized in the boxplot **(B)**. The loading plot **(C)** reports the significant correlation between the concentration of each substance and its importance over PC 1 (*p* < 0.05). The G1 group is represented as red circles in scoreplot **(D)** and boxplot **(E)**.

In the serum metabolome, fifty-seven metabolites were identified (Table 2). Among the identified metabolites, thirteen were significantly different between Groups G0 and G2 according to univariate analysis: glutamate, proline, serine, aspartate, isovalerate, and choline showed a significant reduction, whereas 3-hydroxybutyrate, 3-hydroxyisobutyrate, acetate, succinate, 2,3-butanediol, methanol, and methylsuccinate showed a significant increase. The calculated rPCA model is shown in Panels A–C of Fig. 1. The first principal component (PC1) of its scoreplot, accounting for 66.8% of all of the samples’ variance explained, nicely summarizes the overall differences between samples of Group G0, with low PC1 scores, and samples from Group G2, with high PC1 scores. The loading plot showed that the molecules mostly representative of the G0 group were choline, glutamate, proline, and aspartate, while molecules mostly representative of the G2 samples were 3-hydroxyisobutyrate, 2,3-butanediol, methylsuccinate and methanol. When samples pertaining to Group G1 were projected in this 12 molecules’ space (Fig. 1D,E), they appeared, along PC1, between Groups G0 and G2, with their distance from G0 being 36.3% of the G0–G2 distance.

**Table 2:** Mean values and standard error of the mean (SEM) of metabolite concentrations expressed in µmol/L and their *p* value corrected by the Bonferroni method.

Class	Metabolite	G0	G1	G2	SEM	p value
Amino acids and derivates	Glutamate	49.80^a^	45.00^ab^	40.50^b^	1.61	0.001
	Proline	21.80^a^	21.00^a^	18.70^b^	0.70	0.009
	Serine	20.80^a^	18.20^ab^	15.80^b^	1.17	0.007
	Aspartate	3.17^a^	2.68^ab^	2.12^b^	0.24	0.003
	Lysine	13.90	12.80	12.10	0.60	TS
	Isoleucine	21.50	22.50	24.50	1.02	TS
	Valine	44.50	46.20	52.10	2.53	TS
	Alanine	60.50	57.50	52.30	3.33	TS
	Arginine	54.10	53.60	43.20	4.61	TS
	Leucine	26.30	24.90	28.60	1.44	NS
	Dimethylglycine	0.19	0.18	0.25	0.03	NS
	Glycine	118	109	107	7.41	NS
	Asparagine	10.74	10.59	9.99	0.59	NS
	Glutamine	50.80	54.70	55.30	2.79	NS
	Histidine	16.20	14.90	15.90	0.83	NS
	Methionine	4.13	4.34	4.18	0.30	NS
	Threonine	19.80	19.20	19.60	1.49	NS
	Betaine	6.82	8.11	6.30	0.58	NS
	Phenylalanine	8.00	7.56	7.31	0.60	NS
	Tyrosine	7.75	7.33	6.69	0.45	NS
	Creatine	56.30	59.60	58.00	2.69	NS
	Creatinine	7.69	9.02	7.84	0.64	NS
	Taurine	13.40	12.10	10.90	0.93	NS
	3-Methylhistidine	3.97	3.89	4.06	0.29	NS
	Sarcosine	0.56	0.57	0.51	0.03	NS
	N6-acetyl-lysine	5.87	6.19	5.29	0.39	NS
	2-Aminobutyrate	9.58	9.40	9.29	0.65	NS
Organic acids	3-Hydroxyisobutyrate	3.88^a^	5.56^ab^	7.52^b^	0.53	< 0.0001
	Acetate	170^a^	238^ab^	284^b^	28.13	0.008
	Succinate	1.82^a^	1.78^a^	2.75^b^	0.26	0.028
	Formate	12.31	10.24	9.28	0.86	TS
	Pyruvate	5.11	4.97	4.14	0.36	TS
	Propionate	3.39	4.03	4.23	0.76	NS
	Lactate	223	170	185	29	NS
	Citrate	25.60	27.60	24.40	2.69	NS
	Fumarate	0.79	0.75	0.76	0.06	NS
	2-Hydroxybutyrate	6.33	5.68	6.82	0.79	NS
Alcohols	2,3-Butanediol	0.99^a^	1.47^ab^	3.25^b^	0.55	0.003
	Methanol	3.02^a^	4.83^ab^	9.40^b^	1.76	0.033
	Ethanol	3.36	4.01	16.80	5.48	TS
	Glycerol	12.50	12.00	13.60	1.91	NS
	myo-Inositol	7.81	6.96	5.46	0.80	NS
Carbohydrates	Glucose	943	1009	867	31.73	NS
	Mannose	8.51	9.04	7.65	0.67	NS
	Lactose	11.00	11.40	12.10	1.74	NS
	Gluconate	42.70	28.40	41.80	7.67	NS
Amine and derivates	TMAO	13.00	19.00	18.90	2.30	TS
	Dimethylamine	0.79	0.86	0.94	0.13	NS
Fatty acids	Isovalerate	7.60^a^	6.74^ab^	6.26^b^	0.23	0.001
	Methylsuccinate	0.71^a^	0.80^a^	1.19^b^	0.12	0.008
Ketone bodies	3-Hydroxybutyrate	51.30^a^	74.80^ab^	188.10^b^	25.67	< 0.0001
	Acetone	3.91	6.25	54.10	16.83	TS
Sulfone	Dimethyl sulfone	11.35	9.72	6.74	1.28	TS
Vitamin	Choline	2.03^a^	1.83^ab^	1.23^b^	0.18	0.043
Imidazole	Allantoin	16.50	18.30	15.00	1.11	NS
Nucleoside	Uridine	4.92	4.46	4.86	0.38	NS
Guanidine	Methylguanidine	1.06	1.00	0.94	0.05	NS

*NS* not significant, *TS* trend to significance (0.05 ≤ p value ≤ 0.10).

^a,b^Showed significant differences within rows.

In addition to the 13 significantly different metabolites, 11 seemed to show trends toward significance: lysine, alanine, arginine, formate, pyruvate, and dimethylsulfone were reduced, whereas isoleucine, valine, ethanol, trimethylamine-*N*-oxide (TMAO), and acetone were increased. To define the overall underlying structure, a rPCA model was built through the two-step procedure mentioned above (Supplementary Fig. S1). Samples G0 appeared at low scores along PC1, which represented 56.1% of all of the samples’ variance explained by the model, while samples G2 appeared toward positive values. The molecules mostly representative of the former were dimethyl sulfone, myo-inositol and pyruvate, while those mostly representative of sample G2 were valine and isoleucine. Samples G1, projected over this model, were superimposed with those from Group G0. Interestingly, while samples G2 were not different from samples G0 when the 11 molecules were observed singularly, the difference became significant when their overall trend was observed by rPCA.

### Over representation analysis (ORA)

An ORA was used to gain information about the metabolic alterations that were possibly responsible for changes in the metabolome profile (Table 3). This highlighted that lipid metabolism was significantly (p = 0.016) altered by hyperketonemia. In fact, among the 7 molecules that could be referred to as the metabolism of lipids, 4 appeared to be significantly different between Groups G0 and G2. Studying lipid metabolism in deeper detail, the synthesis of phosphatidylserine seemed to be peculiarly overrepresented, with each of the two molecules observed (choline and serine) significantly altered. Another metabolic pathway influenced by hyperketonemia was glycosaminoglycan metabolism.

**Table 3:** Overrepresentation analysis (ORA) based on the metabolites significantly different between healthy (G0) and hyperketonemic (G2) animals.

Pathway name (reactome’s code)	*p *value	Significantly different metabolites observed	Total molecules observed
Metabolism of lipids (556833)	0.016	4 (3-hydroxybutyrate, acetate, choline, serine)	7 (3-hydroxybutyrate, acetate, choline, serine, formate, glycerol, myo-inositol)
Synthesis of phosphatidylserine (1483101)	0.031	2 (choline, serine)	2 (choline, serine)
Glycosaminoglycan metabolism (1630316)	0.031	2 (acetate, aspartate)	2 (acetate, aspartate)

## Discussion

During the transition period, cows develop NEB status due to a rapid increase in energy demand after the partum, high consumption of glucose by the mammary gland and a corresponding reduction of its availability, possibly causing hypoglycemia^17^. In response to this condition, cows undergo an increase in gluconeogenesis^18^.

The main glucogenic substrates are propionic acid, lactic acid, glycerol and glucogenic amino acids^14^. These substrates are used because sugars and starches are metabolized during ruminal fermentation, while they are common sources of glucose in monogastric species^19^. A deficiency of glucogenic substrates is an important risk factor for the pathogenesis of ketosis^14^. This disease often occurs between the second and seventh weeks postpartum in dairy cows, and subclinical ketosis or hyperketonemia is frequently observed^20^. A better knowledge of healthy and pathologic animals’ metabolome profiles could be useful to understand metabolic alterations associated with BHB increments^9,21^.

In agreement with previous investigations^10^, samples from Group G2 showed NEFA values significantly higher than those from Group G0, confirming the excessive lipid mobilization in bovines during peripartum. An excess of circulating NEFA can induce BHB production through incomplete fatty acid oxidation^5,7^. Oxidation of NEFA may lead to an increase in reactive oxygen species (ROS) in mitochondria due to the respiratory chain. Therefore, the greater concentration of NEFA and BHB may suggest a state of oxidative stress. In addition, NEFA may positively influence ROS production in neutrophils further increasing oxidative stress and influencing the immune response^22–24^. More interestingly, Group G1 showed NEFA values between those of G0 and G2, which were significantly different from both groups. This confirms that cows identified as healthy but with an increment of ketone bodies are indeed switching to a diseased status; consequently, they would need proper management and to be managed separately from healthy animals. However, studies focusing on better management of animals enrolled in the G1 group have not yet been conducted to authors’ knowledge.

In agreement with the literature^11^, in our study, the mobilization of body reserves was represented by an increase in the ketone bodies BHB and acetone in the G2 group, even if only the former was significant, due to a high variance in the latter. BHB is a product derived from muscular amino acid degradation or it can be synthesized from acetyl-CoA. It can be used as an energy source for other tissues, such as the brain and heart^11^. According to Zhang et al. (2017), it can influence the inflammatory response by reducing macrophage function and the production of proinflammatory cytokines.

Ketone bodies can be synthesized from ketogenic amino acids (lysine and leucine) and glucogenic and ketogenic amino acids (isoleucine)^25,26^. However, while isoleucine tended to increase from G0 to G2, lysine tended to show the opposite trend, in agreement with Sun et al. (2014) and Wang et al. (2016). These findings may suggest that lysine could be one of the first amino acids utilized as an energy supply. In addition, in humans, high levels of branched-chain amino acids (BCAAs; isoleucine, leucine, and valine) have been found to be connected to obesity and diabetes, with reports that an increase in BCAAs can lead to insulin resistance and, on the other hand, that higher insulin concentrations can promote protein synthesis^27^. In our study, the increase in BCAA concentrations (isoleucine and valine) in hyperketonemic cows could suggest that hyperketonemic cows may develop progressive insulin resistance proportionally to the BHB concentration. In a recent study on dairy cows, a single-dose duodenal infusion of leucine was not effective in stimulating an insulin response^28^. However, this study examined only six cows in late lactation and evaluated only the response to leucine, which was not significant in our study. Further investigation may therefore be necessary.

Methanol, which was significantly more concentrated in the G2 group, could be derived from methionine metabolism^26^ or from methane during the methane metabolism pathway. However, methionine does not exhibit concentration changes that would suggest an alteration in its metabolism. Methane is produced during ruminal microbial fermentation and is positively related to the mean retention time of particulates and liquid in the rumen^29^. The significant increase that we observed for methanol allows us to speculate that methane was more concentrated in the rumen, produced from other metabolites. As a first confirmation, Yanibada et al. (2020) found in the plasma of dairy cows that dimethylsulfone and formate are inversely related to methane ruminal production. A second confirmation comes from acetate, a fatty acid produced during ruminal fermentation^30^, which was significantly increased in Group G2. This fatty acid is positively related to methanogenesis^31^ and can be used by animals for energetic production when bound to coenzyme A^32^. Furthermore, we should consider that all groups were fed the same total mixed ration (TMR) with no differences in the diet administered.

The increase in methanol can also be related to the increase in ethanol concentration because ethanol can inhibit methanol utilization by microorganisms^33^. Ethanol is a product of anaerobic fermentation in the rumen by yeast and bacteria^34^ that can act as an agonist of the GABA receptor with a consequential inhibitory effect. This metabolite can be used by ruminants for energetic production through its conversion to acetate^35^.

The serum concentration of 2,3-butanediol, significantly higher in Group G2, agrees with the observations by^36^, who described this metabolite as produced from 2-butanone through acetoin in several ketoacidotic conditions. In humans, 3-hydroxyisobutyrate serum levels were also found to be directly correlated with the level of ketoacidosis^37^. Experiments on rats suggested that under ketoacidotic conditions, this molecule is produced from valine^37^. Overall, the increment of methanol, acetate, ethanol, 2,3-butanediol, and 3-hydroxyisobutyrate and the reduction of dimethylsulfone and formate seem to suggest a pathologic increase in ruminal fermentation in hyperketonemic cows.

Choline may be consumed along the metabolic pathway toward glycine synthesis and converted to betaine and then to dimethylglycine. In our study, we observed all of these metabolites, but only choline appeared to be significantly altered by the levels of BHB or hyperketonemia. A more likely fate of choline is therefore its conversion to TMAO, which in our study showed a trend toward an increase and a connection with hyperketonemia. According to Xu et al. (2016), TMAO is a marker of oxidative stress because it reduces glycolysis and enhances β-oxidation of fatty acids. As an alternative involvement of choline in lipid metabolism, Sun et al. (2014) noticed that choline supports fatty acid transport and reduces the risk of hepatic lipidosis. The significant reduction in choline may therefore suggest an alteration of lipid transport with an enhanced risk of developing hepatic lipidosis.

The above pieces of information taken together (NEFA, BHB, choline, and TMAO) could reinforce the idea of oxidative stress due to an enhancement of β-oxidation of fatty acids, and it could, in turn, explain the high risk of hepatic lipidosis in the hyperketonemic group.

Methylsuccinate, significantly increased in Group G2, has been previously related to ketotic conditions^38^, probably in relation to acyl-CoA dehydrogenase activity in the β-oxidation of fatty acids^39^. In accordance with the studies of^40^ and^41^, high values of methylsuccinate in urine can be related to genetic disorders such as acyl-CoA dehydrogenase deficiencies. Methylsuccinate is a metabolite related to succinate, an intermediate of TCA, also significantly increased in Group G2.

In addition, isovaleryl-CoA dehydrogenase is a branched-chain dehydrogenase^42^, which degrades isovaleryl-CoA during the leucine cycle. In the next step, the biotin-dependent enzyme methylcrotonyl-CoA carboxylase (MCC) completes the degradation of isovaleryl-CoA to acetoacetate and acetyl-CoA^43^. Furthermore, other biotin-independent pathways may be involved in leucine catabolism, particularly MCC, which is not available, but they require high energy^43^. Isovaleryl-CoA can be converted into isovalerate, a branched-chain saturated fatty acid. In our study, the isovalerate concentration was significantly decreased in the G2 group, with a trend opposite to that of acetate, as reported recently^44^.

This could suggest that isovaleryl-CoA was metabolized by other pathways that can be biotin-independent, with a loss of energy. As previously reported by^45^, the use of biotin can improve dry matter intake, milk yield, and body energy metabolism due to the gluconeogenesis pathway. Furthermore, biotin supplementation may be useful to improve isovaleryl-CoA metabolism for energy production.

Alanine and serine are glucogenic amino acid substrates for pyruvate synthesis^46^. In our study, alanine showed a tendency toward significance, and the change in the serine concentration was significant. The tendency of these metabolites was a progressive decrease with increasing BHB concentration. Furthermore, serine is an important regulator of glutathione synthesis and it is involved in oxidative stress management^47^. The reduction in serine may suggest an inability of the cows to handle a possible oxidative stress state in the early lactation period.

Pyruvate and its precursors alanine and serine showed an inverse relationship with the BHB concentration. Alanine represents one of the major resources for gluconeogenesis^48^. Overall, this seems to suggest a progressive involvement of pyruvate in gluconeogenesis, with a reduction in its concentration, due to the lack of different substrates in Group G2 compared to Group G0.

The first intermediate of TCA is oxaloacetate, which can be used for pyruvate synthesis. Aspartate is a precursor of oxaloacetate^46^. Although oxaloacetate was not identified in our study, aspartate showed a significant reduction in the G2 group. This finding may suggest a possible reduction in oxaloacetate in the hyperketonemic group due to the decrease in precursors involved in its synthesis.

Glutamate, proline, and arginine are glucogenic amino acids leading to α-ketoglutarate synthesis^46^. All of these amino acids are related to glutamate production^49^. Furthermore, proline and arginine are linked in the metabolic pathway of arginine and proline metabolism. In our study, proline and arginine concentrations decreased in G2. The progressive reduction in proline concentration was in agreement with the study of Wang et al.^16^ on ketotic cows. Proline and its products are components of collagen biosynthesis, which contributes to structure, strength and tissue integrity^50^. Furthermore, proline has been found to act as a weak agonist of the glycine receptor and both *N*-methyl-d-aspartate (NMDA) and non-NMDA ionotropic glutamate receptors. Arginine is a urea cycle amino acid^51^ that participates in nerve signal transduction, and its reduction is related to ketosis^17^. The reduced concentration of these metabolites during the glucogenic process could suggest an influence of the urea cycle, signal transduction and recovery from the inflammatory process in hyperketonemic cows.

Isoleucine and valine can be used to synthesize succinyl-CoA and succinate, two other intermediates of TCA^46^. As previously mentioned, succinate showed a significant increase in G2. Higher concentrations of succinyl-CoA led to a block of citrate synthase and α-ketoglutarate dehydrogenase^52^. The following step is the oxidation of succinate to fumarate through succinate dehydrogenase^53^. This context could lead to a reduction in succinyl-CoA production, an increase in succinate synthesis and its utilization for TCA. However, fumarate concentrations did not show significant changes in the G2 group, as did the succinate concentration, which was fumarate’s precursor in TCA. This result suggests that there was a block in the oxidation of succinate. Succinate dehydrogenase is an enzyme involved in TCA and in the electron transport chain, which is comprised of iron-sulfur protein and flavoprotein (FAD) subunits^52,53^. A block of this enzyme may be due to reduced levels of minerals or altered concentrations of FAD. Furthermore, acyl-CoA-dehydrogenases involved in fatty acid oxidation contain FAD^39^. Further studies specifically focused on this step of TCA may be advantageous to analyze this aspect.

The overrepresentation analysis, applied to the metabolites overall identified and to those significantly different between Groups G0 and G2, highlighted lipid metabolism as significantly (p = 0.016) altered by ketosis. In fact, among the 11 molecules that could be involved in the metabolism of lipids, 4 appeared to be significantly different between Groups G0 and G2. Studying lipid metabolism in greater detail, the synthesis of phosphatidylserine seemed to be particularly represented, with each of the two molecules observed to be significantly altered. Phosphatidylserine is synthesized by facilitating the exchange of l-serine with the choline head group in phosphatidylcholine and with the ethanolamine head group in phosphatidylethanolamine.

Furthermore, glycosaminoglycan metabolism was influenced in the hyperketonemic group. Glycosaminoglycans (GAGs) are long, unbranched polysaccharides containing a repeated disaccharide unit comprised of a hexosamine. GAGs are located primarily in the extracellular matrix (ECM) and on cell membranes. GAGs participate in many important signaling events, such as neuronal growth, inflammation and development^54^.

## Conclusion

Metabolomic analysis through ^1^H-NMR is a useful tool to achieve knowledge about metabolic profiling related to serum β-hydroxybutyrate modifications during the transition period in dairy cows. The metabolic state of our hyperketonemic cows suggests (1) a mobilization of body resources; (2) increased anaerobic fermentation; (3) alteration of lipid metabolism; and (4) a potential oxidative stress state. Furthermore, this metabolic profiling proposes the lack of glucogenic substrates and potential alteration of the electron transport chain are involved in ketosis in dairy cows. These findings indicate a possible alteration of inflammatory and healing processes. This study demonstrates that the metabolomic approach can be considered a significant means to achieve knowledge about dairy cow diseases and their pathogenesis.

## Methods

The Ethics Statement was approved by the Animal Care and Use Committee of the University of Padua (ID number 91/2019—“BovineOmics” Projects). Animal care and procedures were conducted in accordance with the Guide for the Care and Use of Laboratory Animals and Directive 2010/63/EU for animal experiments (National law: D.L. 26/2014). This study was carried out in compliance with the ARRIVE guidelines. Informed consent was obtained from the owners for handling the animals and for the clinical activity of the Veterinary Teaching Hospital, University of Padua.

### Animals

Forty-nine Holstein Friesian dairy cows between 15 and 30 days in milk were enrolled from a single high-yielding dairy farm located in the province of Padua (Italy). These animals were selected among dairy cows used for the experimental design of Fiore et al.^10^. The farm had 600 dairy cows and 400 lactating animals, with a mean production of approximately 10.000 kg/cow/lactation. All animals had a dry period of approximately 60 days, with no streaming-up. The same TMR was used for all enrolled animals (Table 4).

**Table 4:** Total mixed ratio (TMR) and feedstuffs used for all groups (G0, G1, and G2).

Chemical composition of TMR	Dry matter (%)	Feedstuff	Dry matter (%)
Crude protein (CP)	15.2	Alfalfa haylage	27.4
Protein digestible (PD)	12.1	Alfalfa hay	21.2
Protein digested in the small intestine when rumen-fermentable nitrogen is limiting (PDIN)	11.2	Cottonseed meal	7.5
Protein digested in the small intestine when rumen-fermentable energy is limiting (PDIE)	11.3	Concentrate mix:	43.9
Dietary protein undegraded in the rumen but truly digestible in the small intestine (PDIA)	5.2	Corn	41.3
Neutral detergent fiber (NDF)	31.3	Barley	19.3
Acid detergent fiber (ADF)	18.8	Wheat	16.4
Acid detergent lignin (ADL)	2.7	Soybean meal	8.0
Ether extract (EE)	4.6	Molasses	8.0
Ashes (ASH)	7.4	Sodium bicarbonate	2.8
Starch (ST)	24.6	Vitamin D	0.06
Nonstructural carbohydrates (NSC)	41.4	Vitamin A	0.06
Calcium (Ca)	0.8	Vitamin E	0.03
Phosphorus (P)	0.4	Mineral salts	1.0

A clinical visit was performed for each animal by veterinarians at the University of Padua. Animals with signs of clinical disease (metritis, retained placenta, abomasal displacement, milk fever, lameness, or mastitis) on the day of sampling or between parturition and the day of sampling were not accepted for this study. Data about parity, days in milk (DIM), milk yield and body condition score (BCS) with a scale of 1 to 5 points^55^ were recorded. The animals did not show a BCS > 3.5 at parturition or on the day of sampling.

### Experimental design

A cross-sectional experimental design was used. Blood sampling was carried out in the late morning on the day of enrollment during the clinical examination. Samples were collected from the coccygeal vein with a vacutainer system for each enrolled cow. The samples were stored in Venosafe tubes (9 mL; Terumo Venosafe, Leuvel, Belgium) containing Clot Activator.

The Venosafe tubes were refrigerated at 4 °C and transported in a portable freezer (CoolFreeze CFX65 W professional, Dometic, Stockholm, Sweden—minimum temperature −22 °C) at the same constant temperature to the laboratory of the Department of Animal Medicine, Production and Health (MAPS) of the University of Padua (Italy) within 1 h of blood sampling. The samples were centrifuged at 3000 rpm for 10 min (Heraeus Labofouge 400, Thermo Scientific, Milan, Italy). Two aliquots of serum were obtained from each cow and were placed in 1.5 mL Eppendorf tubes. One aliquot was stored at −20 °C for biochemical analysis and the other was stored at −80 °C for metabolomic evaluations.

### Blood analysis and group division

The biochemical analysis was performed using an automatic clinical chemistry analyzer (BT3500 Biotecnica instruments SPA, Rome, Italy). The serum BHB concentration was measured using β-hydroxybutyrate enzymatic kinetics (Randox, Milan, Italy; BHB, mmol/L); NEFA were assessed through the NEFA RX Monza test colorimetric method (Randox, Milan, Italy; NEFA, mEq/L). The glucose concentration was measured using a colorimetric method (Biotecnica Instruments SPA, Rome, Italy).

According to the serum BHB concentration, the animals were divided into three groups (Table 1): Group 0 (G0; 12 healthy animals with 4 primiparous, 2 secondiparous, and 6 pluriparous; BHB ≤ 0.50 mmol/L); Group 1 (G1; 19 healthy animals with 5 primiparous, 3 secondiparous, and 11 pluriparous; 0.51 ≤ BHB < 1.0 mmol/L); and Group 2 (G2; 18 hyperketonemic animals with 2 primiparous, 2 secondiparous, and 14 pluriparous; BHB ≥ 1.0 mmol/L). The BHB in the G2 group ranged from 1.00 mmol/L as the minimum value to 2.77 mmol/L as the maximum value.

### Metabolomic analysis

The metabolomics investigation was carried out through an NMR analysis solution with 10 mM 3-(trimethylsilyl)-propionic-2,2,3,3-d_4_ acid sodium salt (TSP) in D_2_O set at pH 7.00 ± 0.02 by means of 1 M phosphate buffer containing 2 mM NaN_3_. TSP was used as an NMR chemical-shift reference, while NaN_3_ avoided microbial proliferation, as suggested by^56^.

Serum samples were prepared for ^1^H-NMR by thawing and centrifuging 1 mL of each sample for 15 min at 18,630*g* and 4 °C. The supernatant (700 μL) was added to 100 μL of NMR analysis solution. Finally, each sample was centrifuged as previously mentioned.

^1^H-NMR spectra were recorded at 298 K with an AVANCE III spectrometer (Bruker, Milan, Italy) operating at a frequency of 600.13 MHz, equipped with the software Topspin 3.5. According to^56^, the signals from broad resonances originating from large molecules were suppressed by a CPMG filter comprised of 400 echoes with a τ of 400 μs and a 180° pulse of 24 μs for a total filter of 330 ms. The water residual signal was suppressed by means of a presaturation technique. This setting employed the cpmgpr1d sequence, part of the standard pulse sequence library. Each spectrum was acquired by summing 256 transients using 32 K data points over a 7184 Hz spectral window, with an acquisition time of 2.28 s and a relaxation delay of 5 s.

The spectral phase was manually adjusted in Topspin, while the subsequent adjustments were performed in R computational language by means of a script developed in-house^57^. After the removal of the residual water signal, the^1^H-NMR spectra were baseline-corrected by means of peak detection, according to the “rolling ball” principle^58^, implemented in the baseline R package^59^. The signals were assigned by comparing their chemical shift and multiplicity with the Chenomx software library (Chenomx Inc., Canada, ver. 8.3).

The molecules of the first serum sample analyzed were quantified by means of an external standard by taking advantage of the principle of reciprocity^60^. Differences in water content among samples were then taken into consideration by probabilistic quotient normalization^61^. Molecule quantification was performed by means of rectangular integration, considering one of the corresponding signals free from interferences.

### Statistical analysis

Statistical analysis was conducted with R software ver. 4.0.3 computational language^57^. Differences among the main characteristics (NEFA, glucose, BCS, DIM, parity, and milk yield) of the groups were tested through one-way ANOVA using the group as a fixed factor (*p *value < 0.05).

The concentration of each serum metabolite not normally distributed was normalized by Box and Cox transformation^62^. An initial comparison assessing the interactions of parity and DIMs versus groups was performed with a linear mixed model. However, no significant results were found, so it was decided to use only group as a fixed effect. Comparisons between the extreme groups (G0 vs. G2) were then made by t-tests, considering p=0.05 as the limit of significance. A post hoc pairwise comparison among metabolite concentrations was performed using Bonferroni correction. A t-test was also used to evaluate differences between the G0-G1 and G1-G2 groups. A trend for significance was considered for metabolites with 0.05 ≤ p value ≤ 0.10.

Robust principal component analysis (rPCA)^63^ was employed on centered and scaled data of significant metabolites as a means to summarize the structure of the data. rPCA was performed through the PcaHubert algorithm, implemented in the “rrcov” package. First, the algorithm detects outlying samples by computing their distance from the others along and orthogonally to the PCA plane. The optimal number of principal components (PCs) is finally determined. The rPCA model is summarized by a score plot and a correlation plot. The score plot shows the overall structure of the data by showing the samples in the PC space. The second plot shows the molecules that mostly determine the structure of the data by reporting the correlations between the concentration of each molecule and the PCs.

Through the MetaboAnalyst (ver. 5.0) web-based application^64^, the databases of PubChem (https://pubchem.ncbi.nlm.nih.gov/), Human Metabolome Database (HMDB; https://hmdb.ca/metabolites/) and Kyoto Encyclopedia of Genes and Genomes (KEGG; https://www.genome.jp/kegg/) were consulted to obtain a functional interpretation of the metabolites.

Metabolic pathways overrepresentation analysis (ORA) was performed by Fisher’s exact test and by employing Reactome (https://reactome.org) as a pathways’ database.

## Supplementary Information

Supplementary Legends.

Supplementary Figure S1.

## Data Availability

The data presented in this study are available by sending an email to the corresponding author.
