# Supplementary material for: Differences in the serum metabolome profile of dairy cows according to the BHB concentration revealed by proton nuclear magnetic resonance spectroscopy (1H-NMR)
Source: Sci Rep. 2022 Feb 15;12:2525. doi: 10.1038/s41598-022-06507-x (PMC8847571; doi:10.1038/s41598-022-06507-x)
Supplement: Supplementary file 1 — Supplementary Legends. [file 41598_2022_6507_MOESM1_ESM.docx]

**Supplementary Figure S1**. rPCA model built on the space constituted by the concentration of the metabolites with a difference between G0 and G2 tending to significance. In the scoreplot (A), samples from the groups G0 and G2 have been represented with black squares and green triangles, respectively. The samples G1, predicted, are represented with red circles. The wide, empty circles represent the median of each samples’ group. The position of the samples along PC1 is summarized in the boxplot B. The loading plot (C) reports the correlation between the concentration of each substance and its importance over PC 1. Gray bars identify significant correlations (*p* < 0.05).
