# Supplementary figures and images for: Differences in the serum metabolome profile of dairy cows according to the BHB concentration revealed by proton nuclear magnetic resonance spectroscopy (1H-NMR)
Source: Sci Rep. 2022 Feb 15;12:2525. doi: 10.1038/s41598-022-06507-x (PMC8847571; doi:10.1038/s41598-022-06507-x)

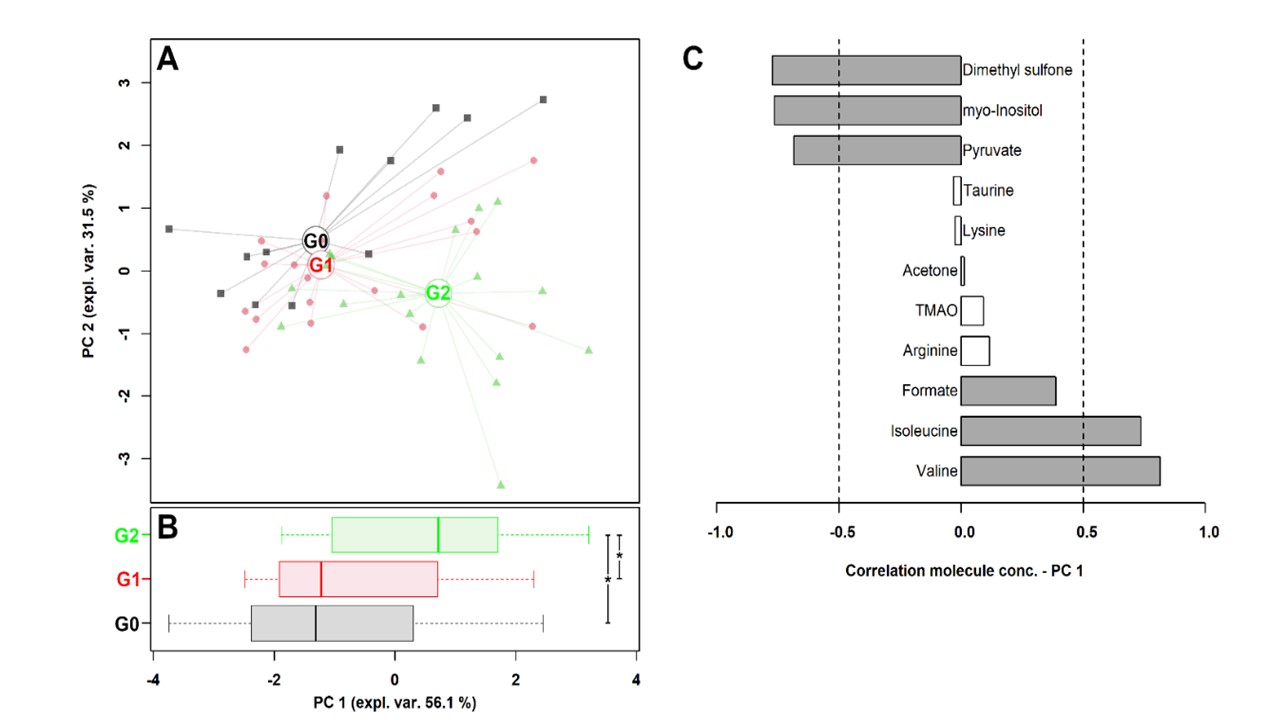

Supplement: Supplementary file 2 — Supplementary Figure S1. [file 41598_2022_6507_MOESM2_ESM.jpg]
